# Circular RNAs: novel actors of Wnt signaling pathway in lung cancer progression

**DOI:** 10.17179/excli2023-6209

**Published:** 2023-07-12

**Authors:** Mina Alimohammadi, Yasaman Gholinezhad, Vahide Mousavi, Samaneh Kahkesh, Malihe Rezaee, Alireza Yaghoobi, Alireza Mafi, Mahmood Araghi

**Affiliations:** 1 Student Research Committee, Department of Immunology, School of Medicine, Shahid Beheshti University of Medical Sciences, Tehran, Iran; 2 Department of Pharmacology, School of Medicine, Shahid Beheshti University of Medical Sciences, Tehran, Iran; 3 School of Medicine, Bushehr University of Medical Sciences, Bushehr, Iran; 4 Faculty of Science, Shahid Chamran University of Ahvaz, Ahvaz, Iran; 5 Department of Clinical Biochemistry, School of Pharmacy and Pharmaceutical Sciences, Isfahan University of Medical Sciences, Isfahan, Iran; 6 Nutrition and Food Security Research Center, Isfahan University of Medical Sciences, Isfahan, Iran; 7 Department of Pathology, School of Medicine, Zanjan University of Medical Sciences, Zanjan, Iran

**Keywords:** CircRNA, Wnt, lung cancer, signaling cascades, mechanism

## Abstract

Circular RNAs (CircRNAs) are a class of regulatory RNA transcripts, which are ubiquitously expressed in eukaryotes. CircRNA dysregulation has been shown to disrupt the interaction of the Wnt/β-catenin pathway, which regulates several biological processes involved in tumorigenesis, thereby contributing to the development and progression of cancer. Interactions of tumor-derived circRNAs with the Wnt/β-catenin signaling pathway provide both clinical diagnostic biomarkers and promising therapeutic targets. In this review, we outlined current evidence on the roles of circRNAs associated with the Wnt/β-catenin pathway in regulating lung cancer formation and development. We believe that our findings will assist in the advancement or establishment of circRNA-based lung cancer therapeutic approaches.

## Abbreviations

**CircRNAs** Circular RNAs

**EMT** Epithelial to mesenchymal transition

**NSCLCs** non-small cell lung carcinomas

**NcRNAs** Non-coding RNAs

**MiRNAs** MicroRNAs

**GPCR** G protein-coupled receptor

**LRP** low-density lipoprotein receptor-related protein

**Fzd** Frizzled

**KLF8** Kruppel-like factor 8

**LUAD** lung adenocarcinoma

**NOVA2** neuro-oncological ventral antigens 2

**PLAGL2** polymorphic adenoma-like protein 2

## Background

Lung cancer is one of the main causes of cancer-induced mortality worldwide. This disease is associated with poor prognosis because of advanced disease presentation, histological subgroup heterogeneity, and a lack of knowledge about cancer pathogenesis (Mridha et al., 2022[75]). Depending on the type of lung cancer, including small cell lung carcinomas (SCLCs) and non-small cell lung carcinomas (NSCLCs), some patients can benefit from drug development to improve their life quality and survival chances, but the vast majority can only be treated with soothing chemotherapy (Lemjabbar-Alaoui et al., 2015[51]). SCLCs are advanced neuroendocrine tumors (NETs) that spread more quickly and are more chemosensitive at first than NSCLCs. NETs contain a variety of tumors with grades ranging from primary typical carcinoid and middle atypical carcinoid to advanced large cell NE carcinoma (LCNEC) (Del Vescovo et al., 2014[16]). On a histopathological level, the disparity among these various parties can be complicated, but it is critical for therapeutic purposes. Aside from SCLC, it has grown increasingly clear in recent decades that NSCLC is a diagnostically and physiologically heterogeneous type of lung cancer that should never be treated like one disorder (Mridha et al., 2022[75], Lemjabbar-Alaoui et al., 2015[51]). The two major subtypes of NSCLC are adenocarcinoma and squamous cell carcinoma (SCC), with another type of carcinoma, large cell carcinoma, lacking clinical characteristics of adeno- or squamous differentiation (Travis et al., 2013[99]). Depending on the subtype, some therapies appear to have different adverse effects in different patients. Recent advances in lung cancer treatment have been achieved by antibody and small molecule-based therapies that target signaling pathways, growth factor receptors, and pro- or anti-tumor genes. Several of these drugs have completed clinical trials (Ray et al., 2010[83]). To develop novel promising therapeutic agents, more understanding of the underlying pathways in lung cancer is required. 

A growing body of evidence suggested that the Wnt pathway is a primary signal transduction pathway involved in lung homeostasis and that abnormal Wnt pathway activation may be involved in lung carcinogenesis (Zhang and Wang, 2020[141]). Intriguingly, although some epigenetic modifications affecting Wnt signaling pathway antagonists are comparable to those observed in other cancers, Wnt pathway variations in NSCLCs are infrequent (Stewart, 2014[93]; Zhang and Wang, 2020[141]). 

Noncoding genes, also known as transcription byproducts or noncoding RNAs (ncRNAs), account for more than 98 % of the human genome (Saw et al., 2021[86]). As whole-genome sequence analysis has progressed, the characteristics of ncRNAs have gradually emerged, and various RNA species, including microRNAs (miRNAs), long ncRNAs (lncRNAs), and circular RNAs (circRNAs), have been identified (Saw et al., 2021[86]). CircRNAs are single-stranded ncRNAs that lack both 5' end caps and 3' end poly (A) tails and are found in almost all organisms (Mafi et al., 2021[69]). CircRNAs have been shown to regulate cancer onset, expansion, and invasion by interacting with Wnt signaling. The circRNA/Wnt axis promotes tumor cell proliferation by targeting several cellular activities in cancer-related genes (Hu et al., 2023[35]). CircRNAs have been linked to a variety of clinicopathological features (Zeng et al., 2018[135]: Liu et al., 2020[62]), suggesting that the circRNA/Wnt axis could play an important role as cancer biomarkers with diagnostic, prognostic, and therapeutic potential.

In this review, we summarized the current studies of the role of crosstalk between circRNAs and the Wnt pathway in the initiation and progression of lung cancer. We also presented the clinical applications of Wnt-associated circRNAs in patients with lung cancer.

## A Brief Overview of Wnt Signaling Pathway: Tumorigenesis and its Role in Lung Cancer

The Wnt signaling pathway is a crucial intracellular pathway that adjusts numerous biological mechanisms including embryonic development, stem-cell maintenance, cell expansion, cell cycle progression, cell death, chemotaxis, and cell adhesion (Nayak et al., 2016[76]; Ng et al., 2019[77]). Aberrant Wnt signaling can lead to the development of many cancers, by affecting the behavior of cancer stem cells (Duchartre et al., 2016[22]). The first Wnt gene was extracted from mouse tumor tissues in 1982. This gene is nearly identical to the *Drosophila* wingless (Wg) gene, which is contributed to the development of wings, fragmentation, and body axis structure. Humans and mice encode 19 Wnt genes, and Drosophila encodes 5 genes (Katoh and Katoh, 2022[45]). Cell-secreted Wnt proteins can induce cellular mechanisms by activating Frizzled (Fzd) membrane proteins, G protein-coupled receptor (GPCR)-linked seven-transmembrane proteins, intracellular proteins, and transcription factors. In addition, Wnt signaling may require the involvement of additional co-receptors, such as the low-density lipoprotein receptor-related protein (LRP) (Katoh and Katoh, 2022[45]). 

Wnt pathway includes two different types of signal transduction pathways (STPs): the canonical (Wnt/β-catenin) and non-canonical (planar cell polarity (PCP) and Wnt/calcium (Wnt/Ca^2+ ^)) (Nayak et al., 2016[76]). The Wnt/β-catenin pathway regulates gene transcription and is negatively affected by the Rnf43 protein. The PCP pathway regulates the cytoskeleton and consequently, the shape of the cell; this pathway can be negatively affected by the Vangl protein. The Wnt/Ca^2+ ^pathway regulates the amount of calcium within the cell (Gajos-Michniewicz and Czyz, 2020[23]).

### The Wnt signaling pathway in tumorigenesis

A deficiency in the canonical or non-canonical STPs can lead to tumorigenesis. The main difference between canonical and non-canonical STP is the presence of β-catenin protein, which is encoded by the CTNNB1 gene. In general, canonical STP has two modes: active and inactive. In the active mode, in the presence of the Wnt ligand, this pathway starts with binding a Wnt ligand to its receptors, Fzd and LRP5/6, which are located in the cell membrane. Then, a multiprotein complex known as signalosome is formed and prevents the activity of the destruction complex, a complex that is active in the absence of Wnt protein and includes Axin, Adenomatous Polyposis Coli (APC), Glycogen Synthase Kinase 3 (GSK3), casein kinase 1 α (CK1α), beta-transducin repeat-containing E3 ubiquitin-protein ligase (β-TrCP), yes-associated protein (YAP), and Transcriptional co-activator with PDZ-binding motif (TAZ). Subsequently, the signalosome is endocytosed into early endosomes, and later, matures into multivesicular bodies. As a result, the β-catenin protein is stabilized and translocated into the nucleus and then binds to TCF/LE, stimulating the activity of the Wnt promoter region (Colozza and Koo, 2021[13]; Zhang and Wang, 2020[141]). In the inactive mode, in the absence of the Wnt ligand, accumulated β-catenin in the cell binds to the destroying complex AXIN and APC. CK1 phosphorylates β-catenin, and then phosphorylated β-catenin is degraded in proteasome by GSK3 (with the help of β-TrCP/YAP/TAZ). Mutilation in the Wnt ligand-dependent or downstream of the ligand-receptor interface can cause cancer-associated Wnt hyperactivation. For example, a mutation in *RNF43* or *CTTB1* genes, APC, AXIN, or other components of the signaling pathway often can cause various cancers including colorectal, gastric, lung, prostate, ovarian, and breast cancer (Parsons et al., 2021[80]). In general, the PCP pathway is started by binding Wnt5A, Wnt7A, and Wnt11 ligands to non-canonical Fzd receptors and ROR2 (RAR-related orphan receptor 2), which are located in the cell membrane, at the same time. Subsequently, Dishevelled (DVL) joins to Dishevelled associated activator of morphogenesis 1 (Daam1), and the DVL-Daam1 complex is built up in the cytoplasm. This process is followed by RAC and RHO (Ras homolog gene family member) activation, and then, c-Jun N-terminal kinases (JNK) and RHO-associated kinase (ROCK) are activated subsequently. JNK modulates cell migration and ROCK plays a role in actin cytoskeleton rearrangement (Gajos-Michniewicz and Czyz, 2020[23]; Duchartre et al., 2016[22]). A mutation in *WNT5A*, *WNT11*, *FZD7*, *VANGL1*, and *VANGL2* can facilitate the migration of cancer cells, and as a result, the malignancy of solid tumors. For example, breast, gastric, colon, colorectal, prostate, ovarian cancer, thyroid and hepatocellular carcinoma, and hematopoietic malignancies can occur as a result of a defect in the PCP pathway (VanderVorst et al., 2018[100]).

Like the previous 2 pathways, Wnt ligands bind to Fzd to start the Wnt/Ca ^2+^ pathway, followed by activation of PLC (Phospholipase C) and then, PKC (protein kinase C). As a result, the concentration of calcium in the cell is increased, followed by triggering the activation of calcium/calmodulin-dependent protein kinase II (CaMKII) and nuclear factor of activated T cells (NFAT), finally leading to transcriptional regulation (Akoumianakis et al., 2022[2]). Several calcium-related factors play a role in tumorigenesis. For instance, intracellular calcium concentration, calcium-related proteins, cell membrane calcium channels, and Wnt/Ca^2+^ pathways are related to tumorigenesis and progression. Especially, Wnt/calcium pathway can lead to melanogenesis, and cutaneous melanoma (Zhang et al., 2022[137]; Dissanayake and Weeraratna, 2008[21]).

### Wnt in lung cancer

Today, lung cancer remains among the most common fatal diseases worldwide; contributing to 11.6 % of total cancers (Lahiri et al., 2023[50]). According to World Health Organization (WHO), NSCLC and SCLC account for 80-85 % and 15 % of all lung cancer cases respectively. It has been demonstrated that a defective Wnt/β-catenin STP can lead to NSCLC. As previously mentioned, Dvl, a phosphoprotein family member, has three isoforms in mammals; Dvl1, Dvl2, and Dvl3. Among them, Dvl1 promotes Wnt/β-catenin signaling overexpression and leads to NSCLC. Several studies have found that Dvl1 expression is higher in NSCLC lung tissue samples than in normal lung tissue samples. Dvl1 cannot be expressed in bronchial and alveolar epithelial cells in normal lung tissue. Moreover, research has shown that Dvl1 expression is also higher in ADC than in SCC, and is higher in stages II-III than in stage I. However, there was no direct correlation with sex, age, or lymphatic metastasis. Daam1 may also play a role in the PCP pathway in lung cancer. Daam1 overexpression is closely related to lung cancer cell metastasis, especially SCLC. PTPs (protein tyrosine phosphatases) and PTKs (protein tyrosine kinases) are responsible for regulating cell migration. PTPN3, a type of PTP, can regulate the expression of the Daam1 protein. A PTPN3 deficiency can result in Daam1 overexpression and, ultimately, lung cancer (Li et al., 2019[54]; Yu et al., 2023[134]).

## CircRNAs: Characterization, Biogenesis and Function

CircRNAs are abundant in many eukaryotic organisms and are implicated in numerous biological processes (Barrett and Salzman, 2016[5]). These unique RNAs are covalent closed, single-stranded transcripts that are derived from pre-mRNA and lack 5' to 3' ends, polarity, and a polyadenylated tail (Qu et al., 2015[82]). They have highly stable molecular structures. These features make them more resistant to RNA exonucleases. They have distinct expression profiles in different tissues and have been linked to morphogenesis and differentiation (Kristensen et al., 2018[48]). Most circRNAs are longer than 200 nt, but their lengths can vary significantly. Nevertheless, some exonic and intronic circRNAs have been demonstrated to be shorter than 200 nt and, in some cases, even shorter than 100 nt. A significant proportion of circRNAs are derived from protein-coding genes, and they often comprise 1-5 exons (Memczak et al., 2013[73]). 

Alternatively, they can originate from non-coding, 3' UTR, 5' UTR, or intronic genomic regions (Memczak et al., 2013[73]). CircRNA generation occurs through a process called Back-splicing of pre-mRNA by spliceosomes, where a 5' acceptor splice site is linked to a 3' donor splice site (Wang and Wang, 2015[109]). Back-splicing, like canonical splicing, is heavily regulated by cis-acting and trans-acting splicing regulatory proteins. Nevertheless, the regulatory mechanism governing back-splicing and its control over circRNA development differentiates from that governing canonical splicing. It is noteworthy that, despite utilizing similar splicing regulatory elements, their activities may vary greatly or even be contrary (Wang and Wang, 2015[109]). Furthermore, compared to the linear RNA canonical splicing, a single genetic locus is capable of generating diverse circRNAs employing the back-splice substitution region as a target (Zhang et al., 2016[140]). CircRNAs may be produced through both canonical and noncanonical splicing processes. CircRNAs can be assigned to four categories based on their origins in different gene sequences, as determined by RNA sequencing: exonic circRNAs (ecircRNAs), circular intronic RNAs (ciRNAs), retained-intron or exon-intron circRNAs (EIciRNAs), and intergenic circRNAs (Memczak et al., 2013[73]). Despite the lack of a precise understanding of the biogenesis mechanism of circRNAs, three models have been suggested for the formation of circRNAs. These models are lariat-driven circularization (exon skipping), intron pairing-driven circularization, and resplicing-driven circularization (Jeck et al., 2013[41]). 

CircRNAs play a crucial role in transcriptional regulation via a variety of mechanisms, including acting as miRNA sponges, RBP sponges, and transcriptional and translational modulators. Additionally, numerous circ-RNAs have been demonstrated to be capable of peptide translation (Granados-Riveron and Aquino-Jarquin, 2016[29]). CircRNAs can compete for miRNA binding sites with their miRNA sponge function, reducing the impact of miRNA regulation processes such as post-transcriptional repression. In comparison to linear miRNA sponges, circRNAs are more stable and more effective. The nucleoplasmic accumulation of EIciRNAs and ciRNAs contained some target miRNA binding sites, and knocking down these circRNAs commonly leads to decreased expression of their parental genes (Zhang et al., 2013[142]). CircRNAs, like several lncRNAs, have been shown to act as scaffolds for RBPs that regulate transcription. While during splicing, circRNAs and their linear homologs may compete for the biosynthesis pathway. Nonetheless, the derived circRNA patterns could enhance the expression of both circRNA and mRNA. Furthermore, certain circRNAs can also modulate protein expression through the sequestration of mRNA translation start sites (Mazloomi et al., 2023[72]).

## The Role of CircRNAs in Lung Cancer

There is currently little understanding of the role of circRNAs in the onset and advancement of cancer. The most common mechanism of circRNA functions in cancer cells is thought to be their role as miRNA sponges (Zhu et al., 2023[148]). MiRNAs have a wide range of roles in cellular processes, such as cellular differentiation, development, proliferation, and apoptosis. The deregulation of these processes mediated by miRNA is a common occurrence in cancer and can play a role in cancer initiation and progression (Mafi et al., 2022[68]). As circRNAs often regulate miRNA function through sponge-like binding, dysregulation in circRNA expression levels could impact their association with tumor-associated miRNAs, highlighting the significant role of circRNAs in the regulation of cancer (Nie et al., 2022[79]; Zhu et al., 2023[148]).

CircRNAs can either promote or inhibit the proliferation, migration, and invasion of lung cancer cells by regulating the activation of the Wnt/β-catenin dependent on the canonical pathway. Upregulated circRNAs in lung cancer cells promote cancer cell proliferation in vitro (Chen et al., 2021[9]). In NSCLC tissues, circRNA_100876 displayed a significant upregulation, when compared to their normal neighboring tissues and was strongly associated with NSCLC metastasis to regional lymph nodes in advanced stages (Yao et al., 2017[127]). The circRNA_100876 can control the expression of MMP-13 by inhibiting the activity of miR-136. This mechanism allows the circRNA to participate in the degradation of the extracellular matrix of chondrocytes (Liu et al., 2016[64]). Because MMP-13 is frequently upregulated in NSCLC, it boosts the probability of metastasis (Yu et al., 2015[133]; Hsu et al., 2006[34]), circRNA_100876 may play a role in the NSCLC cell proliferation, progression, and metastasis by modulating MMP-13 expression via its miRNA sponge function (Yao et al., 2017[127]). According to the survival analysis, NSCLC patients with elevated circRNA_100876 expression had considerably lower survival rates compared to those with decreased expression. This suggests that circRNA_100876 may serve as a possible new prognostic marker for NSCLC (Yao et al., 2017[127]).

## CircRNAs regulation Wnt signaling in Cancer

As previously introduced, studies indicate that the aberrant activation of the Wnt pathway is closely linked to tumorigenesis and tumor metastasis and plays a role in the regulation of epithelial-mesenchymal transition (EMT), the primary mechanism behind tumor metastasis (Clevers and Nusse, 2012[12]). Increasing evidence suggests that miRNAs regulate the process of EMT by interacting with specific target mRNAs of the Wnt/β-catenin signaling in tumors (Song et al., 2015[92]). CircRNAs contain the miRNA targeting site, which can bind to miRNAs. Therefore, circRNAs could perform their functions by regulating the Wnt/β-catenin signaling pathway. Through direct or indirect interaction with the Wnt pathway, circRNAs can either positively or negatively modulate cancer formation, development, and advancement (Xue et al., 2022[121]). Wnt-related circRNAs exhibit atypical expression patterns in malignancies including gastrointestinal, urogenital, and respiratory tracts, as well as musculoskeletal, endocrine, and other types of cancer. Li et al. observed that reducing hsa_circ_000984 levels led to reduced activity of transcription factors such as β-catenin, cyclin D1, and c-myc, implying that hsa_circ_000984 may act as an oncogene by regulating the Wnt/β-catenin pathway. Additionally, they discovered that hsa_circ_000984 may promote NSCLC cell invasion and metastasis by activation of the Wnt/β-catenin signaling pathway (Li et al., 2019[57]). Another study showed that circ_0001730 activates the Wnt/β-catenin pathway through sponging miR-326 and thus leads to growth, invasion, and proliferation in glioblastoma cells (Lu et al., 2019[67]). In the study by Zhu et al. circ_0067934 inhibitsmiR-1324 and leads to activation of the FZD5/Wnt/β-catenin signaling pathway. According to the results of this study, circ_0067934 stimulates invasion, migration, and proliferation in hepatocellular carcinoma (HCC) cells via miR-1324/FZD5/β-catenin signaling axis (Zhu et al., 2018[147]).

## CircRNAs Targeting the Wnt Pathway in Lung Cancer

**Figure 1:**
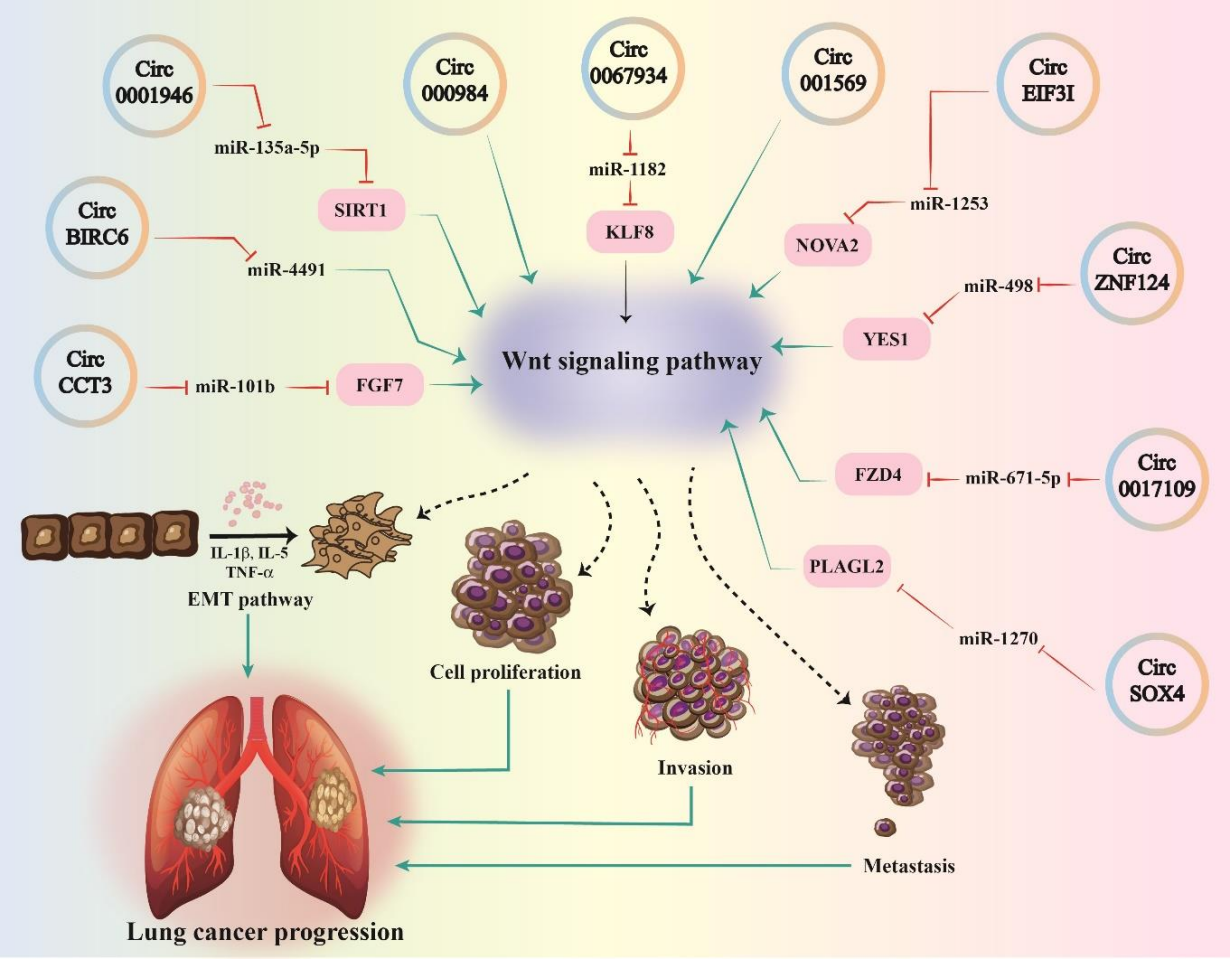
An Illustration of the circRNAs that directly or through specific miRNA sponges activate the Wnt signaling pathway and play a role in the progression of lung cancer.

Recent research has revealed that the Wnt-related circRNAs signaling pathway is overexpressed in lung cancers and is linked to a variety of clinical features. Furthermore, the circRNA/Wnt axis promotes lung cancer cell growth by regulating several cell processes (Figure 1(Fig. 1)). In this section, we will discuss the circRNAs/Wnt axis expression, clinical characteristics, and mechanisms (Table 1(Tab. 1); References in Table 1: Chen et al., 2022[10]; Ding et al., 2018[19]; Gao and Ye, 2020[26]; Gao et al., 2019[27], 2021[25]; Jin et al., 2021[43]; Li et al, 2019[57], 2022[53]; Tian et al., 2017[98]; Wan et al., 2016[101]; Yang et al., 2022[123]; Yao et al., 2019[128][129], 2021[130]; Zhao et al., 2020[143]). This research offers new knowledge about the fundamental processes of cancer development and advancement, allowing researchers to develop new treatment strategies.

### Hsa-Circ_000984

The *CDK6* gene on chromosome 7q generates the circular RNA has_circ_000984 (Memczak et al., 2013[73]). Current studies demonstrated that dysregulation of CDK6 is correlated with NSCLC development and progression (Tadesse et al., 2015[96]), suggesting that has_circ_000984 may have an impact on cancer progression (Xu et al., 2017[119]). Based upon the expression of hsa_circ_000984 in lung cancer cells, multivariate analysis demonstrated that an increased level of hsa_circ_000984 is linked to an unfavorable prognosis and low survival rate in patients with NSCLC (Li et al., 2019[57]). The findings demonstrated that has_circ_000984 is responsible for tumor cell growth, colonization, invasion, and EMT in NSCLC (Li et al., 2019[57]). It has also been shown that depletion of hsa_circ_000984 correlates with raised levels of caspase 3 and caspase 9 proteins and as a result apoptosis in NSCLC can be affected by hsa_circ_000984 (Li et al., 2019[57]). More importantly, hsa_circ_000984 induces its oncogenic property by upregulation of the Wnt/β-catenin signal pathway. This effect occurs by increasing the levels of β-catenin, which causes cell proliferation by activating the transcription of target genes such as cyclin D1 and c-myc, resulting in the development of NSCLC (Li et al., 2019[57]). Thus, this circRNA can provide a new biomarker for predicting prognosis and providing a therapeutic strategy based on molecular methods for NSCLC patients.

### Circ_001569

Circ_001569 was discovered in colorectal cancer. This ncRNA promotes the proliferation and invasion of colorectal cells by sponge miR-145 (Xie et al., 2016[116]). Additionally, high expression of circ_001569 in different malignancies is a common phenomenon. Shen et al. showed that the upregulation of circ_001569 in gastric cancer increased cell proliferation and downregulated cell apoptosis (Shen et al., 2019[89]). In another study, increased levels of circ_001569 are associated with metastasis and poor prognosis in breast cancer (Xu et al., 2019[117]). Also, its role in pancreatic (Shen et al., 2021[90]) and hepatocellular cancer (Liu et al., 2018[61]) has been proven. More investigations revealed that circ_001569 is increased in NSCLC. Indeed, suppressing the expression of circ_001569 is significantly correlated with reduced cell proliferation of NSCLC cells (Ding et al., 2018[19]). Besides, circ_001569 exerts its oncogenic effect in NSCLC by downregulating the miRNA associated with Wnt1 expression, as well as the downstream Wnt/β-catenin signaling pathway, which includes β-catenin and TCF4 (Ding et al., 2018[19]). Finally, overexpression of circ_001569 correlates with TNM staging and low overall survival in NSCLC patients (Ding et al., 2018[19]).

### Circ_0043256

Circ_0043256 is a 483-nucleotide stable circular RNA that is the transcript from chromosome-17 (Zhou et al., 2023[146]). Outcomes derived from RT-qPCR demonstrated that the f (Li et al., 2022[58]). Li et al. also revealed the role of circ_0043256 in the development of NSCLC, which mainly suppressed the proliferation and cell cycle progression of tumor cells (Li et al., 2022[58]). The mechanistic pathway of circ_0043256 in NSCLC has been examined by Tian et al. (2017[98]). They induced the expression of circ_0043256 by treating NSCLC cells with cinnamaldehyde (CA) (Tian et al., 2017[98]). CA is the main component of cinnamon and numerous studies discovered its anticancer property through inhibiting cell proliferation and promoting apoptosis signaling in cancer cells such as in bladder cancer (Aminzadeh et al., 2022[3]), ovarian cancer (Wang et al., 2022[108]), and gastric cancer (Kim, 2022[46]). According to the findings, circ_0043256 was activated by sponge miRNA-1252 and inhibited the Wnt/β-catenin pathway (Tian et al., 2017[98]). One of the targets of miRNA-1252 is the regulation of E3 ubiquitin-protein ligase (ITCH) expression (Tian et al., 2017[98]). ITCH protein has an inhibitory effect on the Wnt/β-catenin signaling pathway and is regulated by circRNA-ITCH (Peng and Wang, 2020[81]). Circ-ITCH acts as a tumor suppressor in cancers like lung cancer, and breast cancer by overexpression of ITCH (Wan et al., 2016[101]; Wang et al., 2019[104]). Additionally, circ_0043256 not only downregulates cell proliferation but also induces cell apoptosis through the upregulation of Bax and decreases the levels of Bcl-2 (Tian et al., 2017[98]). Circ_0043256/miR-1252/ITCH has been proposed as an anti-cancer axis by downregulating the Wnt/β-catenin pathway and increasing apoptosis in NSCLC (Tian et al., 2017[98]). This result can provide new insight into the treatment of lung cancer.

### Circ_CCT3

Circ_CCT3, also known as hsa_circ_0004680, is derived from chaperonin containing TCP1 subunit 3 (CCT3) (Kulcheski et al., 2016[49]). Previous studies indicated the oncogenic role of circ_CCT3 in cancers. Li et al. demonstrated that circ_CCT3 can promote metastasis by regulating Wnt signaling and VEGFA (Li et al., 2020[55]). Circ_CCT3 also increased tumor progression by suppressing miR-613 in pancreatic cancer (Hou et al., 2021[32]). Circ_CCT3 functions in NSCLC by suppressing miRNA-107 (Li et al., 2022[53]). MiRNA-107 has been shown to downregulate the level of vimentin and upregulate the levels of E-cadherin in NSCLC (Li et al., 2022[53]). As a result, the upregulation of circ_CCT3 can induce the EMT pathway by sponging miRNA-107 (Li et al., 2022[53]). On the other hand, the fibroblast growth factor 7 (FGF7) subfamily, a member of the FGF family, is correlated with tumorigenesis in cancers such as cervical (Shang et al., 2019[87]) and lung cancer (Yamayoshi et al., 2004[122]). Liu et al. showed that FGF7 is one of the targets of the Wnt signaling pathway (Liu et al., 2022[66]). In NSCLC, miRNA-107 and FGF7 have an inverse correlation, so upregulation of circ_CCT3 is associated with FGF7 expression by sponging miRNA-107 and eventually regulating the Wnt signaling pathway (Li et al., 2022[53]). Furthermore, the knockdown of circ_CCT3 is prominently associated with Wnt3a gene expression and downregulation of the Wnt signaling pathway (Li et al., 2022[53]).

### Circ_0067934

Circ_0067934 is a member of ncRNAs which is located in chromosomal region 3q26.2 (Xia et al., 2016[115]). Previous studies proved circ_0067934 role as an oncogene in cancers such as ovarian cancer, glioma, and lung cancer (Wang and Li, 2018[102]; Cui et al., 2020[15]; Yin et al., 2022[132]). Circ_0067934 can increase the invasion and migration of NSCLC and decrease cell apoptosis through sponge miRNA-1182 (Zhao et al., 2020[143]). Research demonstrated that miRNA-1182 has anti-cancer activity via inhibition of cell proliferation and invasion in various cancers like bladder, ovarian and hepatic cancer (Hou et al., 2018[33]; Jia et al., 2021[42]; Zhou et al., 2016[144]). MiRNA-1182 could hinder NSCLC progression via the negative effect on the levels of Kruppel-like factor 8 (KLF8) (Zhao et al., 2020[143]). Studies implicated that KLF8 is correlated with different cancers including ovarian, breast, and gastric cancer by promoting cancer cell proliferation, invasion, and metastasis (Cherukunnath et al., 2022[11]; Wang et al., 2011[107]; Mao et al., 2019[70]). The regulatory effect of KLF8 on EMT and Wnt signaling pathways has also been approved (Shi et al., 2015[91], Yang et al., 2012[126]). Furthermore, circ_0067934 upregulation is linked to an increase in the levels of β-catenin, cyclin D1, and c-myc, as well as activation of the Wnt/β-catenin pathway via repress miRNA-1182. As a result, circ_0067934 elevation inhibits the regulatory effect of miRNA-1182 and increases KLF8 levels, leading to the upregulation of EMT and the Wnt/β-catenin pathway, as well as tumor progression in NSCLC patients (Zhao et al., 2020[143]). 

### Circ-BIRC6

CircRNA-baculoviral IAP repeat-containing 6 (circ-BIRC6) is the product of back-splicing of the BIRC6 transcript from chromosome 2 that participates in cell proliferation, colony formation, and invasion in cancers such as hepatocellular carcinoma and bladder cancer (Zhou et al., 2021[145]; Yang et al., 2019[125]). Circ-BIRC6 accelerates the progression of NSCLC by suppressing apoptosis via Bax inhibition and increased Bcl-2 expression (Jin et al., 2021[43]). Furthermore, circ-BIRC6 induces oncogenic outcomes via sponge miR-4491 (Jin et al., 2021[43]). There is a controversy about the role of miR-4491 in the development of NSCLC. Han et al. claimed that miR-4491 is upregulated in NSCLC cells and is associated with cell proliferation and cancer advancement (Han et al., 2021[30]). On the other hand, Jin et al. implicated that miR-4491 has an anti-cancer effect in NSCLC patients by negatively targeting Wnt2B levels (Jin et al., 2021[43]). Nonetheless, inhibiting circ-BIRC6 is associated with decreased levels of Wnt2B and β-catenin via overexpression of miR-4491 and, ultimately, downregulation of the Wnt2B/β-catenin signaling pathway (Jin et al., 2021[43]). This axis results in tumor development, invasion, colony formation, and apoptosis in NSCLC patients and could be a possible molecular target for therapy (Jin et al., 2021[43]).

### Circ-EIF3I

Investigations revealed that circ-EIF3I, also known as hsa_circ_0011385, plays a role in several cancers, including HCC, thyroid cancer, cervical cancer, and pancreatic ductal adenocarcinoma, by increasing cell proliferation, invasion, and metastasis (Ni et al., 2021[78]; Xia et al., 2020[114]; Hu et al., 2022[36]; Wu et al., 2022[111]). The studies also approved the association of circ-EIF3I overexpression with poor prognosis and TNM staging (Chen et al., 2022[10]). The oncogenic regulatory effect of circ-EIF3I is achieved by repressing miR-1253 (Chen et al., 2022[10]). MiR-1253 has anti-cancer properties and has been shown to regulate cell expansion, migration, and metastasis in colon cancer, osteosarcoma, and lung cancer (Yang and Zhang, 2021[124]; Liu et al., 2021[63]; Mo et al., 2021[74]). One of the miR-1253 targets in the inhibition of lung cancer development is neuro-oncological ventral antigens 2 (NOVA2) (Liu et al., 2021[63]), which plays a co and post-transcriptional splicing role in the process of pre-mRNA to mRNA transformation and also participates in the development of neural components and angiogenesis (Angiolini et al., 2019[4]; Mattioli et al., 2020[71]). More investigations demonstrated that increased NOVA2 is associated with cancers such as glioma and colorectal cancer (Li et al., 2019[52]; Gallo et al., 2018[24]). Chen et al. showed that miR-1253 negatively regulates the expression of NOVA2 in lung cancer (Chen et al., 2022[10]). Circ-EIF3I promoted lung cancer progression and apoptosis inhibition through the miR-1253/NOVA2 axis which results in the increased level of β-catenin, c-Myc, and cyclin D1 and upregulation of Wnt/β-catenin pathway (Chen et al., 2022[10]). As a result of the study's findings, it is possible to hypothesize that circ-EIF3I might represent a promising therapeutic target for lung cancer.

### Circ-SOX4

Circ-SOX4 is one of the ncRNAs which is located on chromosome 6 and has been discovered for the first time to be increased in lung adenocarcinoma (LUAD) (Gao and Ye, 2020[26]). Furthermore, experiments revealed that the upregulation of circ-SOX4 is associated with cell proliferation, invasion, and metastasis (Gao and Ye, 2020[26]). According to previous studies, circRNAs perform an oncogenic role by suppressing miRNAs (Xu et al., 2019[118]). Therefore, the knockdown of circ-SOX4 is associated with the upregulation of miR-1270 (Gao and Ye, 2020[26]). The role of miR-1270 has been approved in different cancers such as cervical cancer, thyroid cancer, and breast cancer (Wang et al., 2021[105]; Hu et al., 2022[37]; Yi et al., 2019[131]; Liu et al., 2021[65]). In LUAD, miR-1270 induces anticancer effects by negatively regulating the expression of polymorphic adenoma-like protein 2 (PLAGL2), a zinc finger protein in the PLAG superfamily (Gao and Ye, 2020[26]). This protein can attach to DNA and activate particular gene transcription (Kas et al., 1998[44]). Interestingly, overexpression of β-catenin, CCND1, CDK2, c-MYC, and MMP2 in LUAD has been observed through the upregulation of PLAGL2 (Gao and Ye, 2020[26]). Also, miR-1270 is negatively associated with the EMT pathway by increasing the level of E-cadherin and reducing CD44 and N-cadherin proteins (Gao and Ye, 2020[26]). As a result, circ-SOX4 promotes cell growth and LUAD progression by sponge miR-1270 and overexpression of PLAGL2 by activating the Wnt pathway (Gao and Ye, 2020[26]). These results highlight the role of the circ-SOX4/miR-1270/PLAGL2 axis in the stimulation of Wnt signaling in LUAD and may lead to the development of novel molecular treatment methods for LUAD.

### Circ_0001946

Hsa_circ_0001946 with 145nt is generated from chromosome X (Huang et al., 2019[38]). Circ_0001946 has opposite functions in different cancers. For example, circ_0001946 inhibits cancer development in bladder cancer and glioblastoma (Li and Diao, 2019[56]; Shen et al., 2020[88]). On the other hand, it promotes cell proliferation and the EMT pathway in colorectal cancer (Deng et al., 2020[17]). In LUAD, the circ_0001946 is upregulated and is associated with TNM staging and poor prognosis through elevating cancer cell growth and repressing apoptosis (Yao et al., 2019[129]). More research was conducted to better understand the modulatory mechanism of circ_0001946 on LUAD, and it was discovered that circ_0001946 acts as a molecular sponge for miR-135a-5p (Yao et al., 2019[129]). Expression of miR-135a-5p is associated with various cancers such as breast cancer, glioma, and bladder cancer (Zhang et al., 2022[138]; Diao et al., 2021[18]; Lin et al., 2018[60]). MiR-135a-5p suppressed LUAD progression through the downregulation of sirtuin1 (SIRT1) (Yao et al., 2019[129]). Studies showed that SIRT1 could regulate cell growth, gene expression, and apoptosis in tumors (Knight and Milner, 2012[47]; Blander and Guarente, 2004[7]). Interestingly, SIRT1 participates in cancer prevention and control by regulating the Wnt/β-catenin pathway (Wu et al., 2017[112]). Yao et al. demonstrated that SIRT1 is upregulated in LUAD cells and positively activated the Wnt/β-catenin pathway (Yao et al., 2019[129]). The oncogene circ_0001946 not only was correlated with poor survival in LUAD patients but also promoted LUAD progression by upregulating SIRT1 and activating the Wnt/β-catenin pathway via miR-135a-5p sponging (Yao et al., 2019[129]). Hence, circ 0001946 may be important as an applicable biomarker for the diagnosis or therapy of LUAD.

### Circ_0007059

Circ_0007059 is an anti-oncogenic RNA which is originated from gene ZNF720/chromosome 16 (Hui et al., 2022[39]). More investigation has shown that the higher stage of lung cancer is associated with lower expression of circ_0007059 (Gao et al., 2019[27]). Gao et al. discovered that circ_0007059 overexpression inhibits cell proliferation and promotes apoptosis by increasing the levels of Bax, p53, cleaved-caspase-3, and cyclin D1 (Gao et al., 2019[27]). Furthermore, increased levels of circ_0007059 are associated with the EMT pathway by inhibiting vimentin, Zeb1, and Twist1, and elevating E-cadherin levels via sponge a cancer promoter, miR-378 (Gao et al., 2019[27]). MiR-378 is a regulator of cell growth, metastasis, and angiogenesis in different cancers (Zeng et al., 2017[136]; Wang et al., 2021[103]). In lung cancer, miR-378 promotes cancer invasion and angiogenesis (Ho et al., 2018[31]). Circ_0007059 also inhibits the expression of Wnt3a and β-catenin in cells by repressing miR-378, thereby downregulating the ERK1/2 and Wnt/β-catenin pathway, preventing lung cancer cell proliferation, tumor staging, and metastasis (Gao et al., 2019[27]). 

### Circ_0018414

Circ_0018414 was discovered for the first time to be derived from dickkopf1 (DKK1) on chromosome 10 (Yao et al., 2021[130]). DKK1 is a protein with 266 amino acids that functions as a tumor suppressor in various cancers by competitively attaching its c-terminal domain to Wnt co-receptor-LRP5/6 (Wu et al., 2000[113]; Zorn, 2001[149]). This antagonizing mechanism suppresses the expression of downstream genes, resulting in the inhibition of the Wnt signaling pathway and cancer cell proliferation (Itasaki et al., 2003[40]). Interestingly, overexpression of DKK1 lessened the stemness proteins including Nanog, SOX2, and OCT4. It has been shown that DKK1 levels rise when MiR-6807-3p is inhibited. Therefore, circ_0018414 suppressed LUAD development and improved prognosis in patients via sponging miR-6807-3p and upregulation of DKK1 (Yao et al., 2021[130]). The findings of this study could give a new perspective on the function of circ_0018414 as a tumor suppressor in the treatment of LUAD in the future.

### Circ_0006427

Among miRNA sponges, circ_0006427 is another circRNA that regulates the LUAD progression (Yao et al., 2019[128]). Circ_0006427 is a newly discovered RNA and Sun et al., recently, proved its role in NSCLC as an anti-oncogene through miR-346/VGLL4 pathway (Sun et al., 2022[95]). Circ_0006427 expression is downregulated in LUAD cells and thus regulates lung cancer in another pathway and upregulation of circ_0006427 is negatively linked to TNM stage and metastasis (Yao et al., 2019[128]). Circ_0006427 restrained LUAD cell invasion and migration, increased E-cadherin expression, decreased N-cadherin expression, and suppressed EMT pathway by sponge miR-6783-3p (Yao et al., 2019[128]). Based on experiments, miR-6783-3p is overexpressed in LUAD cells (Yao et al., 2019[128]). DKK1 is a target of miR-6783-3p via the restrain co-receptor LRP5/6. So, circ_0006427 inhibits the Wnt/β-catenin pathway via the miR-6783-3p sponge, allowing DKK1 levels to rise, thereby improving the prognosis and survival of LUAD patients (Yao et al., 2019[128]). The potential of circ 0006427 as a possible therapeutic target for the treatment of LUAD may be clarified by more research.

### Circ_0017109

Circ_0017109 is a lately discovered circRNA with oncogenic properties in NSCLC (Yang et al., 2022[123]). The role of circ_0017109 in LUAD has recently been established via the miR-135b-3p/TOX3 axis (Wang et al., 2022[110]). Overexpression of circ_0017109 is correlated with TNM staging, metastasis, and poor overall survival in NSCLC. Overturned circ_0017109 induces cell proliferation and Bcl-2 expression but decreases the levels of cleaved-caspase-3 which result in the inhibition of apoptosis (Yang et al., 2022[123]). Circ_0017109 exerts its effect on NSCLC through sponge miR-671-5p (Yang et al., 2022[123]). Previous literature demonstrated that miR-671-5p plays a regulatory role in different cancers such as papillary thyroid carcinoma and breast cancer (Wang et al., 2021[106]; Tan et al., 2019[97]). MiR-671-5p inhibits cell proliferation and cancer progression in NSCLC by targeting FZD4, a member of Frizzled genes family that participate in the Wnt pathway to regulate tissue development and cell proliferation (Yang et al., 2022[123]). Yang et al. found that overexpression of circ_0017109 increased β-catenin, c-myc, and cyclin D1 expression in cells by upregulating FZD4 (Yang et al., 2022[123]). In addition, circ_0017109 promotes NSCLC progression by suppressing miR-671-5p. Knocking down miR-671-5p causes FZD4 overexpression and, as a result, upregulation of the Wnt/β-catenin pathway, which causes tumor growth and metastasis and worsens the prognosis of NSCLC patients (Yang et al., 2022[123]). Based on this regulatory network, circ 0017109 may be a promising indicator for the diagnosis and prognosis of NSCLC patients.

### Circ-ITCH

Circ-ITCH is a well-known circRNA that is located on the 20q11.22 chromosome (Xu et al., 2018[120]). Previous studies have shown the role of circ-ITCH in biological pathways including cell proliferation, differentiation, apoptosis, inflammation, drug resistance, and cancer regulation (Su et al., 2022[94]). ITCH as a member of the E3 ubiquitin ligases family (Bernassola et al., 2008[6]) is associated with cancer development by modulating the expression levels of p53, p73, and p63 and also adjusting the Wnt pathway via degrading Dvl and FZD4 (Aki et al., 2015[1]; Bernassola et al., 2008[6]). Circ-ITCH is downregulated in lung cancer and is negatively associated with the TNM stage as well as survival (Li et al., 2019[59]). Circ-ITCH can inhibit lung cancer cell proliferation by binding to the 3′-UTR of miR-7 and miR-214 as oncogenic miRNA and inhibiting their inhibitory mechanism on ITCH, thereby suppressing Wnt/β-catenin signaling and lung cancer progression (Wan et al., 2016[101]). Therefore, circ-ITCH with its function in the miR-7, miR-214/ ITCH axis can be considered as an RNA-based NSCLC diagnostic and therapeutic target.

### Circ-ZNF124

circ_0017348, known as circ-ZNF124, is another member of the circRNA family with a regulatory role in lung cancer which is originally from zinc finger protein 124 (Ding et al., 2018[20]). Recent research has demonstrated that circ-ZNF124 is overexpressed in NSCLC (Gao et al., 2021[25]). Gao et al. discovered that circ-ZNF124 has an oncogenic effect on NSCLC by promoting cell proliferation and invasion, whereas repressed levels of circ-ZNF124 keep cells in the G0/G1 phase and cause more apoptosis (Gao et al., 2021[25]). Circ-ZNF124 plays a role in NSCLC via sponge miR-498 (Gao et al., 2021[25]). According to previous research, miR-498 regulates cell proliferation, invasion, metastasis, prognosis, and drug resistance in cancer (Zhang et al., 2019[139]; Cao et al., 2022[8]; Cong et al., 2015[14]). More investigations found that miR-498 refrains NSCLC development by downregulation of YES proto-oncogene 1 (YES1) (Ding et al., 2018[20]). It has been demonstrated that YES1 expression in cancers controls cell proliferation and invasion (Garmendia et al., 2022[28]). Additionally, YES1 is upregulated in lung cancer and is associated with poor prognosis (Sato et al., 2022[85]; Redin et al., 2022[84]). Interestingly, the upregulation of circ-ZNF124 ascends β-catenin and c-Myc levels in NSCLC cells (Gao et al., 2021[25]). Also, YES1 regulates NSCLC progression through Wnt/β-catenin pathway upregulation (Gao et al., 2021[25]). Researchers discovered that circ-ZNF124 regulates NSCLC development by repressing miR-498 and upregulating YES1, resulting in tumor growth and increased recruitment of the Wnt/β-catenin pathway, thus downregulating Circ-ZNF124 might suppress NSCLC development partly via miR-498/YES1 modulatory complex and inactivating the Wnt/β-catenin signaling pathway (Gao et al., 2021[25]). 

## Conclusion and Future Perspectives

**Figure 2:**
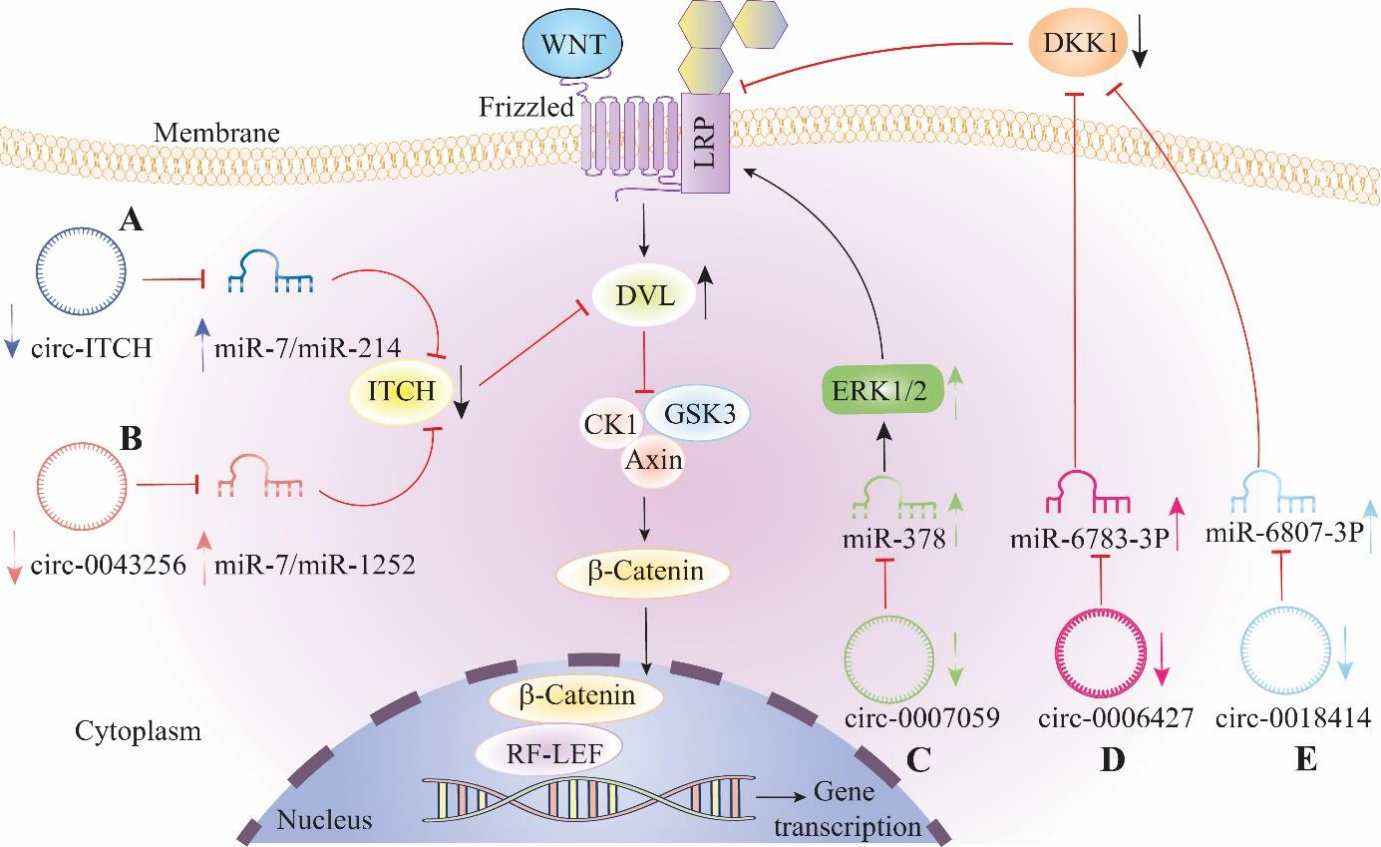
The downregulation of circular RNAs promotes lung cancer progression through the upregulation of the Wnt/β-catenin pathway. A, B, reduced levels of circ-ITCH and circ-0043256 promotes aggregation of miR-7/miR-214 and miR-7/miR-1252 respectively, which causes ITCH reduction. Increased DVL as a result of ITCH downregulation, stabilizes β-catenin in cells and induces the Wnt/β-catenin pathway. C, inhibitory effect on miR-378 declines, by downregulation of circ-0007059 which causes ERK1/2 rise. ERK1/2 activates the Wnt/β-catenin pathway by interaction with the LRP protein. D, E, a drop of circ-0006427 and circ-18414 expression in lung cancer cells, results in upregulation of miR-6783-3P and miR-6807-3P respectively, which depletes DKK1 expression and Wnt/β-catenin pathway activation. Abbreviations: ITCH, Itchy E3 Ubiquitin Protein Ligase; LRP, Low-density lipoprotein receptor-related protein; DVL, Dishevelled; ERK1/2, extracellular signal-regulated kinases 1/2; DKK1, dickkopf1

Despite advancements in diagnostic and therapeutic approaches, common relapse, chemoresistance, and metastatic potential have continued to be the main causes of poor prognosis in several malignancies including lung cancer. CircRNAs have the potential to lead to the development of new targeted therapies as well as biomarkers for early detection. CircRNAs influence cancer cell growth, migration, and metastasis via multiple pre- and post-transcriptional pathways. The most common method is to act as a miRNA sponge, allowing them to increase the expression of their targets. According to the research, circ-RNAs can also regulate gene and protein expression, as well as modify the levels of some signaling-related elements, such as those that encode peptides and amino acids. Furthermore, circRNAs play a role as "enhancers," controlling the functions of certain proteins while maintaining their expression profiles. Because of their regulatory mechanisms, circRNAs are useful in lung cancer research. Among the various signaling pathways that regulate the impacts of circRNAs on biological functions, the Wnt/β-catenin pathway is found in several cancers and plays a fundamental role in lung cancer development and advancement. CircRNAs influence the Wnt/β-catenin signaling pathway through interactions with signaling pathway components such as genetic and epigenetic factors (Figure 2(Fig. 2)). Furthermore, circRNAs regulate the Wnt/β-catenin signaling pathway via both the traditional and alternative circumvent pathways, including the Rspo/Lgr4 and CTNNBIP1 pathways. The Wnt/β-catenin signaling pathway is negatively regulated by CTNNB1, ITCH, APC, and CBL, while it is activated by FZD, β-catenin, and Dvl. Recent findings suggest that circRNAs play an important role in lung cancer pathogenesis via the Wnt/β-catenin signaling pathway. The interactions between circRNAs and the Wnt/β-catenin signaling pathway may assist in the discovery of new treatments and screening predictors. The mechanisms underlying such interactions should be investigated further to advance combined therapies. 

It can be inferred from this review that circRNAs can be used as a promising anti-lung cancer therapy in light of their indisputable modulatory roles in the development of lung cancer. In other words, the circRNA-mediated Wnt/β-catenin pathway can be a crucial supplement to the medical treatment of lung cancer. Undoubtedly, the roles and targets of some Wnt/β-catenin pathway-related circ-RNAs are currently unknown and must be investigated further in the future. More research is needed to determine the underlying processes that lead to cancer formation and progression, allowing for the development of more effective predictive and therapeutic biomarkers. 

## Notes

Mina Alimohammadi and Yasaman Gholinezhad contributed equally as first author.

Alireza Mafi and Mahmood Araghi (Department of Pathology, School of Medicine, Zanjan University of Medical Sciences, Zanjan, Iran; E-mail: araghimah@gmail.com) contributed equally as corresponding author.

## Declaration

### Acknowledgements

Not applicable.

### Availability of data and material

Not applicable.

### Funding

No specific source of funding is associated with this work.

### Availability of data and material

Not applicable.

### Author contributions

The authors confirm contribution to this paper as follows; Mina Alimohammadi: Writing and review manuscript. Yasaman Gholinezhad, Vahide Mousavi and Samaneh Kahkesh: Data collection, writing, and draft preparation. Yasaman Gholinezhad: Contributed to draw figures and the table in the manuscript. Malihe Rezaee and AlirezaYaghoobi: Data collection and writing. Alireza Mafi: Conceptualization, investigation, database searching and revising the manuscript. Alireza Mafi, Mahmood Araghi, and Malihe Rezaee: Review, editing, validation and project administration. All authors read and approved the final version of manuscript for submission.

### Ethics approval and consent to participate

Not applicable.

### Consent for publication

Not applicable.

### Competing interests

The authors declare no conflict of interest.

## Figures and Tables

**Table 1:**
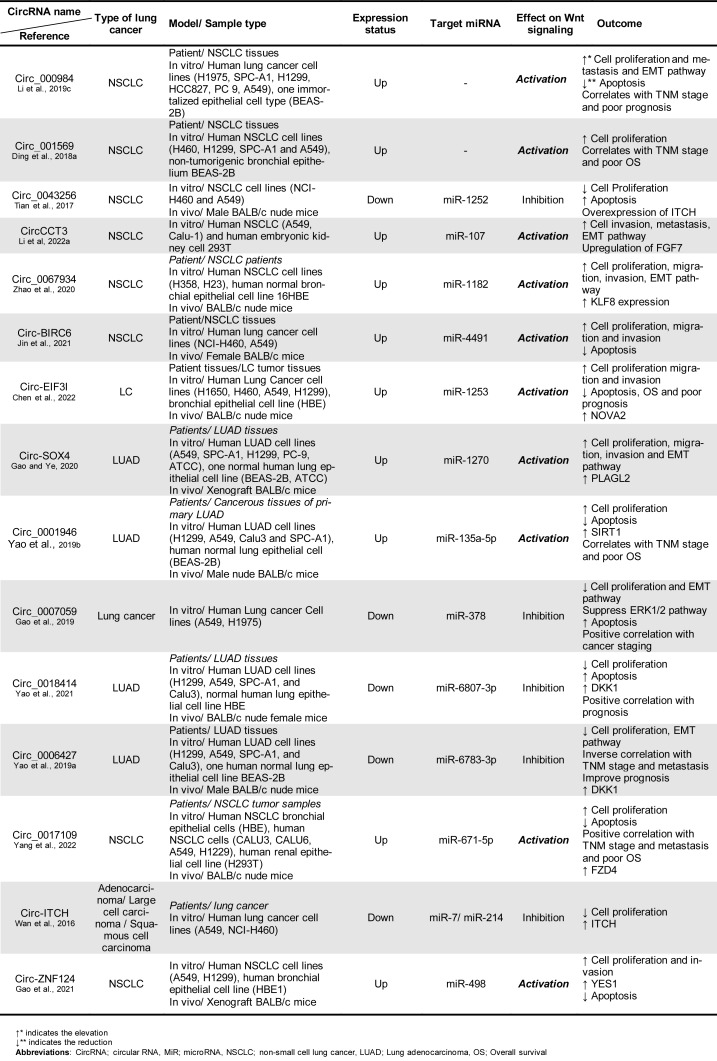
CircRNAs that are involved in the Wnt signaling pathway in lung cancer
